# Human Leukocyte Antigen (HLA) and Islet Autoantibodies Are Tools to Characterize Type 1 Diabetes in Arab Countries: Emphasis on Kuwait

**DOI:** 10.1155/2019/9786078

**Published:** 2019-11-20

**Authors:** Mohamed Jahromi, Ebaa Al-Ozairi

**Affiliations:** ^1^Sahatoke Center, Manama, Bahrain; ^2^Clinical Research, Medical Division, Kuwait; ^3^Dasman Diabetes Institute, Kuwait; ^4^Medical School, Kuwait University, Kuwait

## Abstract

The incidence rate of type 1 diabetes in Kuwait had been increasing exponentially and has doubled in children ≤ 14 years old within almost two decades. Therefore, there is a dire need for a careful systematic familial cohort study. Several immunogenetic factors affect the pathogenesis of the disease. The human leukocyte antigen (HLA) accounts for the major genetic susceptibility to the disease. The triggering agents initiate disease onset by type 1 destruction of pancreatic *β*-cells. Both HLA and anti-islet antibodies can be used to characterize, predict susceptibility to the disease, innovate, or delay the *β*-cell destruction. Evidence from prospective longitudinal studies suggested that the underlying disease process represents a continuum that begins before the symptoms are clinically evident. Autoimmunity of the functional pancreatic *β*-cells results in symptomatic type 1 diabetes and lifelong insulin dependence. The autoantibodies against glutamic acid decarboxylase (GADA), insulinoma antigen-2 (IA-2A), insulin (IAA), and zinc transporter-8 (ZnT-8A) comprise the most reliable biomarkers for type 1 diabetes in both children and adults. Although Kuwait is the second among the top 10 countries with a high incidence rate of type 1 diabetes, there have been no proper diagnostic and prediction tools as per the World Health Organization. The Kuwaiti Type 1 Diabetes Study (KADS) was initiated to understand the disease pathogenesis as well as the HLA and anti-islet autoantibody profile of type 1 diabetes in Kuwait. Understanding the disease sequela in a homogenous gene pool and highly consanguineous population of Kuwaitis could help solve the challenges and pathogenesis, as well as hasten the prevention, of type 1 diabetes.

## 1. Introduction

The incidence of type 1 diabetes (MIM #222100) continues to surge despite several therapeutic advances and has long been noticed to be highly variable among countries. In 2017, the incidence rate varied by 803-fold, with 64.2/100,000 in Finland [1] and 0.08/100,000 in Papua New Guinea [2] (Table 1). Interestingly, the same was observed between countries with comparable health care systems; for example, there was a 12.6-fold variation in incidence rates between Sardinia (54.4/100,000) [1] and Lombardia (4.4/100,000) [1] in Italy. China is another country where there is a 12-fold variation among regions (0.13–1.61/100,000) [3]. The reason for this ethnoracial difference is not precisely known.

Historically, type 1 diabetes has been most prevalent in populations of European origin but is becoming more frequent in other ethnic groups [4]. The Arab league comprises 22 countries and accounts for only 5% of the total world population, but it contributes heavily to the increasing global burden of type 1 diabetes, with 60,000 cases reported in children ≤ 14 years old [5]. According to a recent report, Kuwait is the second of the top 10 countries with a high incidence rate of type 1 diabetes (Table 1) [6, 7]. Moreover, the incidence in children ≤ 14 years old has grown from 20.1/100,000 [8] to 44.9/100,000 in only two decades [6]. The disease's rising incidence in Kuwait might be due to rapid lifestyle changes, such as sedentary lifestyle, changes in breastfeeding practices, type 1 deficiency caused by greater hygienic standards, and low vitamin D levels, which is highly prevalent in the region despite the sunshine [9]. Meanwhile, rates of consanguinity and endogamous marriages in Kuwait are quite high at 22.5% to 64.3%.

Localizing genes and novel mutations in complex diseases have proven to be successful in such populations [10]. Given these facts, there is a dire need for a careful systematic study on type 1 diabetes in Kuwait. According to the literature, the Kuwaiti Type 1 Diabetes Study (KADS) is a familial case/control study, using nuclear family data to estimate case and control marker allele frequencies and diabetes-related autoantibodies. In families ascertained for the presence of an affected child (case), the parental marker alleles not transmitted to the affected child are used as control alleles. KADS screens Kuwaiti T1D patients and their first-degree relatives (parents, sibling, and offspring) for human leukocyte antigen (HLA) class I and class II genes using the next-generation sequencing (NGS) HLA-typing. KADS is a unique systematic study in the Arab population aimed at finding out immunogenetic markers of T1D in the Kuwaiti population and staging the preclinical phase of the disease. Undoubtedly, such studies can explain the rapid rise of this silent killer disease in the region and will add the missing gap of knowledge in understanding the pathogenesis of the disease.

**Figure 1:**
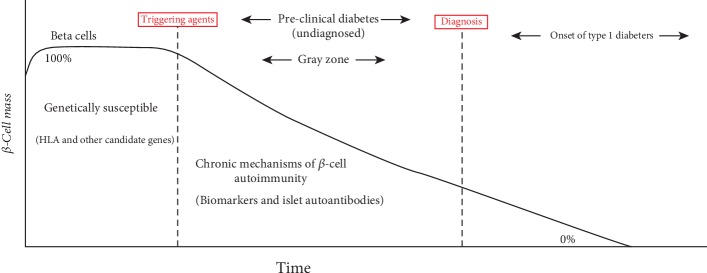
Natural history of type 1 diabetes. During the undiagnosed phase (gray zone), fundamental biological reactions can occur leading to the next phase by which the disease is diagnosed and no islet cell is available to produce indigenous insulin. Figure is adapted from reference with modification.

The natural history of type 1 diabetes was initially proposed over 25 years ago, when both genetic susceptibility and triggering agents (i.e., environmental factors) were found to lead to immune-mediated destruction of pancreatic *β*-cells and loss of *β*-cell function [11]. The disease progresses through a preclinical phase (Figure 1), which can be identified by autoimmunity markers and glucose intolerance arising from further loss of *β*-cell function, and ultimately culminates with clinical signs and symptoms of diabetes [11–13]. Tremendous biochemical and biological reactions might have occurred before *β*-cell autoimmunity. These processes need to be identified before the immune system irreversibly destroys substantial amounts of *β*-cells. However, the progression rate from the preclinical phase of disease [14, 15] to the onset of *β*-cell autoimmunity and symptomatic disease is variable, lasting from months to years [11, 16]. Based on the Finland Diabetes Prediction and Prevention [17], the German BABY-DIAB [18], the International Type 1 Diabetes Trial Network [19], the Diabetes Autoimmunity Study in the Young [20], and The Environmental Determinants of Diabetes in the Young [21] studies, islet autoantibodies can first appear very early in life and predict type 1 diabetes. Consequently, KADS is established to set up a proper diagnostic and prediction tool for type 1 diabetes in Kuwait, based on characterization of the Kuwaiti type 1 diabetes HLA risk and the anti-islet autoantibody profile.

Recently, different phases of type 1 diabetes etiology have been endorsed internationally [14, 15, 22–25]. The preclinical phase was divided into two: normoglycemia and dysglycemia [23]. Furthermore, Insel and colleagues have recognized three different phases of human type 1 diabetes, which allow for interventions designed to delay and, ultimately, prevent the onset of clinical symptoms. However, genetic susceptibility and triggering factors that were accounted for in these current phases were based almost solely on American or European studies.

### 1.1. Genetic Susceptibility: HLA

Genetic susceptibility to type 1 diabetes is determined by polymorphisms/mutations in human genes [26–31]. More than 40 genes are known to influence the progression of type 1 diabetes [32]. Different HLA genotype patterns result in diverse rates of type 1 diabetes among populations [33] and have the greatest impact compared to any other genes. The associations of type 1 diabetes with HLA class II DR, DQ, and DP have been shown to vary among different populations and ethnicities (Table 2) [34–39]. For example, the high-risk HLA haplotypes in Caucasian populations, DRB1∗03:01-DQB1∗02:01 and DRB∗04:01-DQB1∗03:02, were found to be low in incidence in Japan and Southeast Asia; instead, the susceptibility HLA haplotypes in Japanese and Korean populations were DRB1∗04:05-DQB1∗04:01 and DRB1∗09:01-DQB1∗03:03 [38]. In Arab populations (i.e., Bahrainis, Lebanese, and Tunisians), DRB1∗03:01-DQB1∗02:01 was reported [37] (Table 3). In African Americans, the assessment of HLA risk differs significantly from that of other characterized populations; both the DRB1∗07:01 and DRB1∗03:03 were high-risk haplotypes when DQA1∗03:01-DQB1∗02:01 was included [39]. Interestingly, in African Americans, the DRB1∗07:01-DQA1∗02:01-DQB1∗02:01g haplotype was protective against type 1 diabetes risk in European-derived cases but increased the type 1 diabetes risk in African-derived cases [39]. These studies conducted in homogeneous ethnic groups and comparing HLA DR-DQ-DP haplotypes offer evidence to link the risk of developing T1D and specific HLA-DP alleles. More importantly, these studies provide evidence that distribution of DP alleles varies depending on the ethnic group studied [40]. Table 2 summarizes the classification of HLA-DR in different populations and their diabetes risk level [30, 31, 33–35, 37, 41–51].

### 1.2. HLA Alleles versus Haplotypes

HLA genes are not transmitted randomly from the parent to the offspring, with solid linkage disequilibrium between A, C, B, DR, and DQ alleles, i.e., haplotypes [31, 35, 37, 51–59]. However, T1D susceptibility haplotypes exist in a limited number. For instance, in Finland which has the highest incidence of T1D globally, only 37 different HLA haplotypes have been identified among diabetic children who had either a parent or a sibling with T1D and another 18 haplotypes in children with a first-degree relative who does not have T1D [54].

### 1.3. HLA and Type 1 Diabetes in Arabs

There are only a few HLA studies conducted in Arab countries that compare their contribution to the rise of T1D globally (Table 2) [3, 37, 48–60]. Most available studies have not used systematic HLA research standards. They have discussed HLA association randomly on either allele-based or haplotypes [37, 48–60]. Others are quite out of date and have been performed serologically [58]. Nevertheless, these studies have taken the first steps to elucidate genetic risk factors in the Arab population [37, 57, 58, 60]. The hallmark of HLA susceptibility is, however, considered from the haplotype point of view [31, 33, 34, 43, 44, 51–55]. Reported Arab T1D HLA studies were tabulated in Table 3 [49, 50, 55–60], which highlights the dearth need for systematic HLA studies in these populations. A recent meta-analysis published in 2015 analyzed 23,333 articles, of which only 30 were based on an Arab population. These studies mainly discussed genetic susceptibility of T1D related to HLA-DR or DQ alleles but not haplotype configurations [61]. Hamzeh et al. reported that Arab HLA indicate that 80% of patients with T1D are carriers of DR3 or DR4. In addition, HLA-DR3/DR4 is reported in 13%-75% patients with T1D, which present the highest diabetes risk [61].

Furthermore, it has been suggested that the presence of DR9 haplotype is an important factor in the low-rate T1D within the Japanese population [35, 53, 62]. In fact, variation in HLA-DR locus in HLA haplotypes in heterogenetic populations may in part explain the differences in T1D worldwide. However, this variation is not fully understood, since only limited comparisons of HLA haplotypes between populations are available [35, 43, 52].

In fact, the same is true in some neighboring countries in the MENA region like Iran and Turkey. For example, in Iran, they have reported that HLA DRB1∗03:01/DQA1∗05:01/DQB1∗02:01 are risk factors [63]. Another interesting finding in Iran found a correlation with HLA gender specificity and age at onset. Sayad's group reported that HLA DRB1∗04:01, DQB1∗03:02 alleles, and DRB1∗04:01-DQB1∗03:02 haplotypes were significantly more common in male T1D patients compared to female patients, while DRB1∗03:01, DRB1∗15:01, DQB1∗06:01 alleles, DQB1∗03:01/05:01 genotype, DRB1∗03:01-DQB1∗02:01, and DRB1∗15:01-DQB1∗06:01 haplotypes were significantly greater in the female T1D cohort than males. The same team has also reported that age at onset has a significant role in susceptibility to T1D among the latter HLA haplotypes [64]. Conversely, in Turkey, available data confirms similar trends in the distribution of T1D HLA susceptibility genes seen in other Caucasian populations [65].

**Figure 2:**
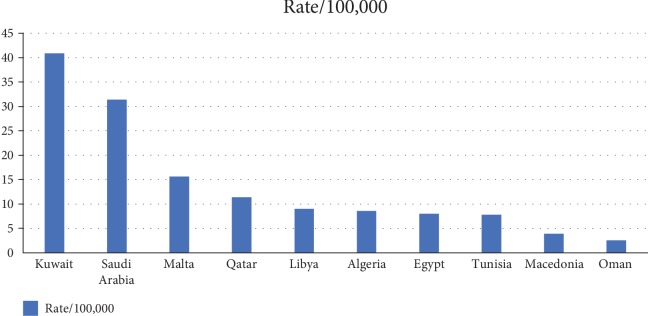
The reported rates of incidents of type 1 diabetes in Arab countries. There is 16.4-fold increase in all Arab countries. Interestingly, the same increase is obvious among petroleum-rich GCC countries, Kuwait, Saudi Arabia, Qatar, and Oman (IDF and Shaltout et al. 2016).

An inadequate number of studies have reported the association between HLA and type 1 diabetes among the neighboring Gulf Cooperation Council (GCC) countries (Figure 2). This is important because the type 1 diabetes rates vary among the GCC countries despite similar geography, culture, and socioeconomic conditions.

### 1.4. Prediction and Diagnosis of Type 1 Diabetes: Anti-islet Autoantibodies

The initial immunofluorescence identification of islet cell antibodies (ICA) in 1974 [66, 67] was a key step in recognizing type 1 diabetes as an immune-mediated disease. The ICA can identify any antibody that binds to human islet tissues in a nonspecific manner with immunofluorescent techniques [66, 67]. However, the ICA assay is difficult to standardize because it is operator-dependent, varies according to the quality of the human pancreatic tissue used as a substrate, and recognizes heterogeneous antibodies that vary among individuals. The ICA is considered a composite of specific anti*β*-cell antibodies, several of which have now been characterized at the molecular level [68], i.e., IAA, GAD, IA-2, and ZnT-8.

Anti*β*-cell antibodies can be used to study the natural history of the preclinical phase of type 1 diabetes, identify individuals at increased risk of diabetes development, and select high-risk individuals for trials on immune intervention. They are also used to distinguish type 1 diabetes from nontype 1 diabetes. The risk of developing clinical disease increases dramatically with an increase in the number of antibodies; it increased to 70-90% in the presence of two and three antibodies [68]. In Caucasian populations, more than 90% of childhood type 1 diabetes were classified in association with HLA class II genes [16]. Islet autoantibodies are involved in the type 1 response, and their types and numbers can help predict [11, 12, 16] and classify [14, 15, 22–25] type 1 diabetes. There is a dearth amount of information about autoantibodies in type 1 diabetes in Arab populations (Table 4). Majority of the present studies are case control and investigated the presence of only GADA and IA2 [49, 50, 69–73]. There are interesting studies correlating gene polymorphisms and the ICA, IA2, and GADA to the susceptibility to type 1 diabetes in the Kuwaiti population [49, 50]. These findings may reflect variation in assay standardization, number of autoantibodies measured, variation in population studied, or existence of other forms of non-immune-mediated diabetes, idiopathic (type 1B). The current incomplete understanding of autoantibody profiles of type 1 diabetes in the Arab population gives emphasis on the urgent need of an international scientific community to study the nature of such a massive increase of type 1 diabetes in the Arab population, e.g., Kuwait. KADS is the first study in the Arab population to characterize type 1 diabetes according to autoantibody profiles and HLA typing. Of course, all our future studies will be based on the latter characterization. We will be able to target neoepitopes which can delay *β*-cell immunity, intervene eventually, and cure this nasty disease.

### 1.5. Insulin Autoantibodies (IAA)

IAAs were described by Palmer et al. in 1983 in insulin-naïve patients with new-onset diabetes [78]. IAAs are very important because they are often the first autoantibodies that can be detected in early childhood. One example of an IAA is proinsulin, which is the only anti-islet antibody expressed almost exclusively in *β*-cells [14] and corresponds to the specific targeting of *β*-cells by the T-cells infiltrating the pancreatic islets. The very high frequency of IAA found in young children upon diabetes onset implied that loss of tolerance to proinsulin was common in those who progress rapidly into the disease [11, 14, 75]. IAAs were also found in a nonobese diabetic mouse model of spontaneous type 1 diabetes [13]; this further supports the role of proinsulin as a primary autoantigen, which is usually the first islet autoantibody to be detected. It is especially common (>70%) in childhood diabetes and is less frequently detected after adolescence.

High-affinity IAAs are generally more predictive of type 1 diabetes and were found to be associated with the disease appearance at a young age, subsequent progression to multiple autoantibody positivity, and binding to human insulin A chain residues 8–13 [14]. In first-degree relatives of type 1 diabetes patients, IAA may be found in more than 90% of children below five years of age but in only half of young adults aged 15 to 21 years. It has the highest positive predictive value among all the islet autoantibodies [13].

### 1.6. Autoantibodies to Glutamate Decarboxylase (GADA)

The next major autoantigen to be identified in the 1990s was a 65 kDa isoform of glutamate decarboxylase (GAD65) [79]. GADA is found in almost 80% of people with type 1 diabetes at all ages and is the characteristic marker of type 1 diabetes in adults [16]. GAD is found in neurons and the pancreas, where it is involved in synthesizing gamma-aminobutyric acid (GABA), which regulates the function of *β*-cells via paracrine and autocrine signaling [79, 80]. GADA is also detected in certain neurologic disorders, indicating that GAD is not diabetes-specific [81].

Critical epitope clusters which appear early in the GADA response have been identified, and the affinity and epitope specificity of the antibody response predict disease progression [82]. In the early stages of diabetes-associated GAD65 autoimmunity, the GADA-recognized epitopes are predominantly located in the middle region of the protein. However, at later stages, these may extend to the N-terminus regions [79]. GADA can also provide one of the critical definitions of Latent Autoimmune Diabetes in Adults (LADA) [13, 16, 20, 82, 83].

### 1.7. Insulinoma Autoantibodies (IA-2A)

Two tryptic digest fragments of islet antigens from type 1 diabetes patients were characterized in 1995 [68]. One was a 40 kDa fragment from the intracellular portion of a tyrosine phosphatase-like protein (*PTPRN* gene) and is now referred to as IA-2ic or ICA512ic [84]. IA-2As are almost always detected with other islet autoantibodies and are very specific for type 1 diabetes [16]. The other 37 kDa tryptic fragment was identified as the IA-2-related protein IA-2*β* or phogrin [84]. Since almost all autoantibodies that react with IA-2*β* also react with IA-2, IA-2*β* autoantibodies are not routinely used by clinical laboratories as a first-line test but may be of particular value for identifying individuals at high risk of disease progression [16]. The critical epitope regions or residues for IA-2A and IA-2*β* antibodies have been defined, and their hierarchy of risk for future type 1 diabetes has been described [85]. Subreactivity to the IA-2*β* protein was strongly associated with progression to diabetes within five years [86]. Autoantibodies to IA-2 are present in up to 80% of children and adolescents upon type 1 diabetes diagnoses [21–23, 86].

### 1.8. Antibodies to the Zinc Transporter-8 (ZnT-8A)

ZnT-8, also known as SLC30A8, is a 35–40 kDa member of the solute carrier- (SLC-) 30A subfamily, which belongs to the CDF family of proteins. It is expressed by pancreatic *β*-cells and alpha-cells, B cells, and adipocytes and is known to play a role in zinc transport. The ZnT-8 appears to transport zinc from the cytosol into secretory vesicles, which, in the case of *β*-cells, provides a necessary component for proper insulin processing and granule storage [87]. The discovery of ZnT-8 was the result of a bioinformatic strategy to define new candidate autoantigens.

ZnT-8A may emerge several years before the disease onset, but it typically appears later than the IAA or GADA [16, 22, 68, 82]. The prevalence of ZnT8-A was as high as 80% among children 12–16 years old [68, 87, 88]. ZnT-8As are virtually absent among Gold Medalist (patients with >50 years of diabetes) [83, 88]. The principal epitope targeted by the ZnT8A is influenced by a single amino acid at position 325, which is encoded as arginine, tryptophan, or glutamine by different polymorphic variants of the ZnT-8-encoding gene *SLC30A-8* [68, 87]. The autoimmunity directed against the COOH-terminal region of ZnT-8 is of particular prognostic significance; in particular, ZnT-8A-positive children who were homozygous for either arginine or tryptophan at position 325 (*SLC30A-8*), rs13266634, were found to have the greatest risk of type 1 diabetes progression compared to heterozygotes [89]. Genome-wide association studies demonstrated a strong association of type 2 diabetes with another SNP in the same position (i.e., rs16889462) that encodes glutamine, although this is rare [90].

The ZnT-8A isoform that is largely confined to pancreatic *β*-cells [68, 87] had been proposed as an independent immune marker of type 1 diabetes [13, 16, 20, 68, 87]. Upon testing ICA-seropositive individuals using four autoantibody standards, ZnT-8 was found in 26% of type 1 diabetes subjects who were classified as autoantibody-negative based on the existing markers GADA, IA2A, and IAA. The combined measurement of ZnT-8A, GADA, IA2A, and IAA increased the autoimmunity detection rates to 98% at disease onset [16, 68, 84, 87, 91]. This resembles self-governing of ZnT-8 as an independent diabetes predictor autoantigen [16, 68, 84, 87, 91]. However, the existence of samples with high ICA but no autoantibody indicates the need to evaluate other islet antibodies.

### 1.9. Autoantibody Profile in the Pathogenesis of Type 1 Diabetes

The risk of progression varies with antibody response intensity; those with higher antibody titers are more likely to progress to clinical disease. Another factor that appears to influence progression of *β*-cell damage is the age at which autoimmunity develops. Months before the actual onset of the disease, IAA is the initial autoantibody that develops, followed by GADA [11, 16].

Autoantibodies against GAD, IA-2, IAA, and ZnT-8 are the most reliable biomarkers for type 1 diabetes in both children and adults [11, 16, 22, 68, 74, 82, 87] and are currently the only biomarkers that can distinguish LADA from phenotypically type 2 diabetes [13, 20, 82, 83, 88]. Because the frequency of autoantibodies upon the diagnosis of childhood type 1 diabetes depends on age, GADA is, by far, the most common in LADA, whereas GADA and IAA are the best markers for childhood diabetes [16, 68, 82, 83]. Multiple autoantibody positivity had been shown to be more common in childhood diabetes than in adult-onset diabetes and has a high predictive value for childhood type 1 diabetes [5, 16, 82, 83]. Results on the use of autoantibodies to predict diabetes in adults have been inconsistent, and autoantibody levels were reported to cause heterogeneity in LADA [83]. Reports indicated that the phenotype of diabetes was more of type 1 in individuals with high levels of autoantibodies and more of type 2 diabetes in individuals with low levels of autoantibody positivity [83, 92].

Autoantibody levels are well known to fluctuate, and transient autoantibody positivity in LADA has been reported to affect the GAD [93], IA-2 [94], IAA [83, 92], and ZnT-8 phenotypes [68, 92]. Currently, anti-islet autoantibodies are considered as immune-signatures of pancreatic *β*-cell autoimmunity during the preclinical phase of the disease [14, 15, 22–25]. Therefore, autoantibody detection is another important step in integrating immunologic data in the KADS, to identify the type 1 diabetes risk profile, especially when using the screening algorithm for relatives of affected cases. In fact, the current phase of characterizing type 1 diabetes in this cohort study of a Kuwaiti population is integration of autoantibodies. A systematic longitudinal follow-up of the high-risk relatives of these patients might unravel certain important issues that developed prior to, and during, different stages of *β*-cell autoimmunity, leading to their destruction. Individuals with two or more positive autoantibodies are candidates for prevention immunotherapy trials.

The appearance of anti-islet autoantibodies may not be the causes, but the consequences, of *β*-cell autoimmunity. The entire process of *β*-cell injury and autoimmunity is thought to transpire during the undiagnosed preclinical episode and upon initiation by triggering agents, which are not yet well-determined.

There are international workshops and proficiency agendas to advance and standardize the assays used for measurement of islet autoantibodies. For example, the Diabetes Antibody Standardization Program (DASP) uses blinded sets of control and patient sera to assess and improve the comparability of GADA, IA-2A, IA, and ZnT8A measurements among laboratories [95]. To adhere with DASP standards, where their autoantibody measurements are being carried out, we will collaborate with the Barbara Davis Center for Childhood Diabetes, USA, to set up our own system and participate DASP thereafter.

## 2. Discussion

Diabetes is a multifactorial disease caused by destruction of pancreatic islet *β*-cells. In our type 1 diabetes population, characterization and identification of the HLA haplotype and pancreatic islet autoantibodies as the present biomarkers for *β*-cell destruction will enable us to develop a scientifically sound prediction algorithm. Screening of first-degree relatives of type 1 diabetes patients can help predict the family members who are at risk of acquiring the disease and improve the management. There might be a therapeutic value in earlier interventions, when there are greater amounts of functioning *β*-cells to preserve, rather than at the clinical phase of diagnosis, when many *β*-cells have been destroyed or damaged.

Type 1 diabetes in Kuwait has an exponentially increasing incidence rate [6] and has not been well-diagnosed. There is paucity of reports on HLA as the major genetic susceptibility predictor and on anti-islet autoantibodies in Kuwaiti individuals with type 1 diabetes. Novel concepts on the rate and degree of *β*-cell loss throughout the natural history of the disease have been put forward to aid in explaining the disease etiology. There is no systematic study from the Arab population on HLA and anti-islet autoantibody profiling so far, and it is essential to have such a study in Arab countries to improve clinical care and add missing part of global type 1 diabetes studies.

## Figures and Tables

**Table 1:** Global ranking of countries as per their rate of type 1 diabetes in children < 15 years old.

Ranking	Country	Rates/100,000
01	Finland	57.2
02	Kuwait	44.5
03	Sweden	39.5
04	Saudi Arabia	33.5
05	Norway	29.8
06	United Kingdom	25.9
07	Ireland	24.3
08	United States of America	23.7
09	Denmark	23.0
10	Australia	22.5
11	Canada	21.7
12	Georgia	18.0
13	Poland	17.3
14	Czech Republic	17.2
15	Puerto Rico	16.8
16	Malta	15.6
17	Belgium	15.4
18	Portugal	13.2
19	France	12.7
20	Qatar	11.4
21	Sudan	10.1
22	Switzerland	9.2
23	Croatia	9.1
24	Libya Arab Jamahiriya	9.0
25	Algeria	8.6
26	Uruguay	8.3
27	Egypt	8.0
28	Brazil	7.7
29	Tunisia	7.3
30	Romania	5.4
31	Georgia	4.6
32	India	4.2
33	Macedonia	3.9
34	Taiwan	3.8
35	Iran	3.7
36	Antigua and Barbuda	3.5
37	Jordon	3.2
38	Oman	2.5
39	Japan	2.4
40	Barbados	2.0
41	Mexico	1.5
42	Uzbekistan	1.2
43	Tajikistan	1.2
44	Paraguay	0.9
45	Zambia	0.8
46	China	0.6
47	Peru	0.5
48	Ethiopia	0.3
49	Papua New Guinea	0.1
50	Venezuela	0.1

Source: IDF Atlas 2017.

**Table 2:** Classification of HLA-DR alleles and their risk level.

HLA-DR	*DQA1*	*DQB1*	*DRB1*	Susceptibility	Populations
*DR2*	01:02	06:02	15:01	Protective	Almost all
*DR2*	01:02	05:02	16:01	Moderate risk	Caucasians
*DR2*	01:03	06:01	15:02	Neutral	Caucasians
*DR3*	05:01	02:01	03:01	High risk	Caucasians, Koreans
*DR4*	03:01	03:02	04:01	High risk	Caucasians
*DR4*	03:01	03:02	04:02	Moderate risk	Caucasians
*DR4*	03:01	03:02	04:03	Neutral	Caucasians
*DR4*	03:01	03:02	04:04	Moderate risk	Caucasians
*DR4*	03:01	03:02	04:05	High risk	Caucasians
*DR4*	03:01	03:01	04:01	Neutral	Caucasians
*DR4*	03:01	03:03	04:01	Neutral	Caucasians
*DR4* ^∗^	04:05	03:03	04:01	High risk	Japanese, Koreans
*DR7*	02:01	03:03	07:01	Protective	Caucasians
*DR6*	01:01	05:03	04:01	Protective	Caucasians
*DR8* ^∗^	08:02	03:01	03:02	High risk	Japanese
*DR9* ^∗^	09:01	03:00	03:03	High risk	Japanese, Koreans
*DR13* ^∗^	13:02	01:02	06:04	High risk	Japanese

^∗^ They are only found in Asians and not in Caucasians.

**Table 3:** Classification of HLA-DR alleles and their risk level in Arab populations.

*HLA*				Susceptibility	Populations
*DR*	*DQA1*	*DQB1*	*DRB1*		
*DR3*	05:01	02:01	03:01	High risk	Bahraini, Kuwaiti, Egyptian, and Tunisian
*DR4*	03:01	03:02	04:05	High risk	Saudi Arabia, Algerian
*DR2*	01:02	06:02	15:01	Neutral	Saudi Arabia, Algerian

Some studies have discussed HLA haplotype rather than allelic variations. Either whole studies or parts which were based on allelic variations were not included in this table.

**Table 4:** Number of anti-islet autoantibody measured in different Arab population studies.

IAA	IA2	GAD	ZnT8	References
✓	✓	✓	✓	[74, 75]
✓	✓	✓	—	[76, 77]
—	✓	✓	—	[49, 50, 69–73]

Although IAA is more frequent in children, it has not been included in majority of studies.
